# Distinct Characteristics of Rye and Wheat Breads Impact on Their *in Vitro* Gastric Disintegration and *in Vivo* Glucose and Insulin Responses

**DOI:** 10.3390/foods5020024

**Published:** 2016-03-25

**Authors:** Emilia Nordlund, Kati Katina, Hannu Mykkänen, Kaisa Poutanen

**Affiliations:** 1 VTT Technical Research Centre of Finland Ltd, 02150 Espoo, Finland; Kaisa.Poutanen@vtt.fi; 2 Department of Food and Environmental sciences, University of Helsinki, 00014 Helsinki, Finland; kati.katina@helsinki.fi; 3 Department of Clinical Nutrition, Food and Health Research Centre, School of Public Health and Clinical Nutrition, University of Eastern Finland, 70211 Kuopio, Finland; Hannu.mykkanen@uef.fi

**Keywords:** rye, wheat, bread, sourdough, *in vitro* digestion, particle size, insulin response, glucose response

## Abstract

Disintegration of rye and wheat breads during *in vitro* gastric digestion and its relation to the postprandial glucose and insulin responses of the breads was studied. Breads with distinct composition and texture characteristics were prepared with refined or wholegrain wheat and rye flour by using either straight dough or sourdough process. After chewing and gastric digestion *in vitro*, 100% wholemeal and refined rye breads prepared by sourdough method were disintegrated to a much lower extent than the wheat breads, having more bread digesta particles with size over 2 or 3 mm. Microstructure of the digesta particles of rye sourdough bread revealed more aggregated and less degraded starch granules when compared to refined wheat bread. The postprandial insulin responses, but not those of glucose, to the 100% rye breads made with sourdough method were lower than the responses to the refined wheat bread. Addition of gluten or bran in rye sourdough bread increased insulin response. PCA (Principal Component Analysis) analysis confirmed that the insulin response had a negative correlation with the number of larger particles after *in vitro* digestion as well as amount of soluble fiber and sourdough process. Since the high relative proportion of large sized particles after chewing and *in vitro* gastric digestion was associated with low postprandial insulin responses, the analysis of structural disintegration *in vitro* is proposed as a complementary tool in predicting postprandial physiology.

## 1. Introduction

Diets containing rapidly absorbing carbohydrates and low in dietary fiber (DF) are associated with increased risk of type 2 diabetes [1,2], whereas consumption of whole grain cereal foods reduces the risk of type 2 diabetes and heart disease, possibly partly via the effects on insulin metabolism [3,4]. Starchy foods like breakfast cereals, standard wheat breads and potato products result in high glycemic responses, whereas intact cereal grains, pasta and dense breads produce lower responses [5,6]. Thus the form of cereal-based starch-rich food products is important for their postprandial responses.

Cereal products have unique solid foam structure, which is formed during processing [7]. Regular refined wheat flour based solid foam structures (e.g., bread) have very porous structure and thus low density, while acidic or fiber enriched solid foam structures (typical, e.g., for rye products) have high density [8]. Wheat based solid foams, such as bread, are based on continuous gluten network, which provides viscoelastic network trapping gas inside the product. Presence of non-digestible carbohydrates, especially soluble arabinoxylan and beta-glucan, results in higher viscosity, macro density and hardness. In 100% rye bread, protein cannot form a continuous network and elastic dough similar to wheat protein [9], and arabinoxylan is the main water-binding substance having larger effect than protein on dough rheology and gas holding properties. Besides compositional differences of wheat and rye flour, lactic acid bacteria fermentation process generally used in rye bread making results in dense structure and altered starch properties and a high number of starch-protein interactions [10]. The distinct structures of rye and wheat breads have been related to starch digestibility and postprandial glucose metabolism [11]. In refined wheat bread, highly gelatinized starch and porous structure are predicted to result in rapid degradation of starch in the small intestine and rapid rise of blood glucose and insulin levels. Rye and also wheat-based bread prepared by fermentation with lactic acid bacteria, *i.e*., sourdough technology, have reported to show reduced GI values [12]. The reduced postprandial responses of rye sourdough breads are expected to be attributed to biochemical factors of rye [13,14,15] and acidity induced solubilization of DF, acidity-mediated reduction of starch digestion and stepwise degradation of protein phase of the foam [10,11,12], but the mechanisms are not completely revealed.

To predict the postprandial glucose metabolism of cereal products, the rate of starch digestion has been investigated by *in vitro* hydrolysis using alimentary amylolytic enzymes [10,12,16,17,18]. However, none of these *in vitro* methods took into account the disintegration and particle size of the products during gastric digestion, which is known to influence the rate of gastric emptying *in vivo* [19,20]. Hernot *et al.* [20] studied the influence of dough processing step of whole meal flours on *in vitro* digestion of starch, but the impact of end product structure was not examined. Hence, the role of food disintegration during gastric digestion in regulating postprandial glycemia is not known. In the present work the target was to show how different bread types disintegrate during oral and simulated gastric phase, and whether the particle size after gastric phase simulation can in part predict glycemic and insulinemic responses *in vivo*. For that, mechanical, structural and biochemical properties of various types of rye and wheat breads, their particle size after the *in vitro* gastric digestion, and *in vivo* glucose and insulin responses were analyzed. A multivariate analysis method was applied to study the relationships between bread characteristics, gastric phase particle size and *in vivo* responses of breads.

## 2. Materials and Methods

### 2.1. Breads

Commercial wholemeal wheat flour (ash content 1.8% d.w), wholemeal rye flour, refined wheat flour (ash content 0.6% d.w), refined rye flour, wheat and rye bran (Melia Ltd, Raisio, Finland) were used for baking the breads. The commercial coarse wheat bran was further ground to obtain fine particle size (6% >750, 47% >355, and 78% >132 μm).

The reference bread (Bread 1) was a commercial refined wheat bread (Table 1). Bread 2 was a commercial wholemeal sour rye bread made with finely milled flour. These breads, commonly used in Finland, were received from two Finnish bakeries (Fazer Bakeries Ltd and Vaasan & Vaasan Oy). The experimental breads baked for the study were: Bread 3, 100% wholemeal rye bread made with sourdough method; Bread 4, 70%:30% wholemeal rye bread with rye bran (70%:30% flour:bran ratio) made with sourdough method; Breads 5 and 6, 100% refined rye bread made with sourdough method (pan and flat bread); Bread 7, 100% refined rye bread (flat) with added gluten made with sourdough method; Bread 8, 60%:40% wholemeal rye/wheat bread made with straight dough method; Bread 9, 60%:40% wholemeal wheat/wheat bread made with straight dough method; and Bread 10, wheat bread with fermented bran (33% supplementation level) made with straight dough method. Hence, Breads 2–7 were prepared with sourdough method and the rest of the breads with straight dough method. Breads 6 and 7 were baked as flat bread and all the other breads were baked as standard pan breads. Recipes and process for experimental breads are presented as supplementary data (Tables S1 and S2).

### 2.2. Chemical Composition, Acidity and Volume of Breads

The amount of available starch was determined by an enzymatic kit (Megazyme method, [21]). Sugars were analyzed after extraction with water for 15 min at 50 °C by the high performance liquid chromatography HPLC-method of Tenkanen *et al.* [22]. The available carbohydrate content was defined as the sum of available starch and sugars. DF was determined as described by Asp *et al.* [23], protein was analyzed by the Kjeldahl method [24], and fat was analyzed using the Fat in Flour-Mojonnier method (AOAC (Association of Official Analytical Chemists) official methods of analysis (2000, no. 922.06 “fat in flour”.) no. 922.06). Acidity of breads was determined by measuring the pH value and total titratable acidity [25]. Specific volume (mL/g) of the fresh breads was determined by the rapeseed displacement method.

### 2.3. Postprandial Glucose and Insulin Responses

The postprandial glucose and insulin responses of the breads were tested in five different experiments each including refined wheat bread as the reference and Breads 1–3. The subjects for Breads 1 and 2 were men and women (*n* = 17), aged 22–37 year, BMI 19.2–26.7 kg/m^2^, all had normal glucose tolerance [26]. The subjects for Breads 3–10 were 12 females aged 64 years (57–72), body weight 68.3 ± 8.4 kg (mean, SD) and with normal glucose tolerance. The meal tests were performed in the morning after the subjects had fasted for 12–15 h. The meal contained 50 g available carbohydrates from the test bread and 40 g cucumber and 3 dL non-caloric orange drink. From the time when the subjects started to eat, altogether 8 blood samples were drawn (15, 30, 45, 60, 90, 120, 150 and 180 min) for the determination of blood glucose and insulin concentrations. The breads were served in a random order with a minimum of a one-week interval. Plasma glucose was analyzed with the enzymatic photometric method (Granutest 100; Merck, Damstadt, Germany) with use of a Kone Specific Clinical Analyzer (Kone Ltd, Espoo, Finland), and serum insulin was analyzed by radioimmunoassay (Phadaseph Insulin RIA 100; Pharmacia Diagnostica, Uppsala, Sweden). Glucose and insulin responses of the Breads 6–10 were determined specifically for this work, whereas the data for Breads 1 and 2 were from Leinonen *et al.* [26], and the data for Breads 3–5 from Juntunen *et al.* [11]. The postprandial incremental blood glucose and insulin areas over 180 min (AUC) were calculated for the test breads and compared to the respective areas for the refined wheat bread in each series. The mean AUC value of each bread was used in PCA analysis.

### 2.4. In Vitro Digestion Method

The digestion method for the particle-size analysis was performed according to the *in vitro* method by Grandfeldt *et al.* [17]. An equivalent amount of available carbohydrate (1 g of starch and sugars on the basis of analyzed data) of the breads was chewed *in vivo* for 15 s by four subjects and the masticated residues were expectorated into a beaker containing 50 mg pepsin (Activity ≥ 2000 FIP-U/g, Merck, Darmstadt, Germany) in 6 mL of 0.05 M NaK phosphate buffer (containing 0.4 g/L NaCl) adjusted to pH 1.5 with HCl. Then the subjects rinsed their mouth with 10 mL of phosphate buffer, (pH 6.9) for 15 s and expectorated the rinsing solution into the beaker. The pH of the suspension was adjusted to 1.5 with HCl. The suspension was incubated for 30 min (37 °C, 400 rpm). Samples for the particle size analysis were removed at this stage.

### 2.5. Digesta Particle Size and Microscopy Analysis

**Figure 1:**
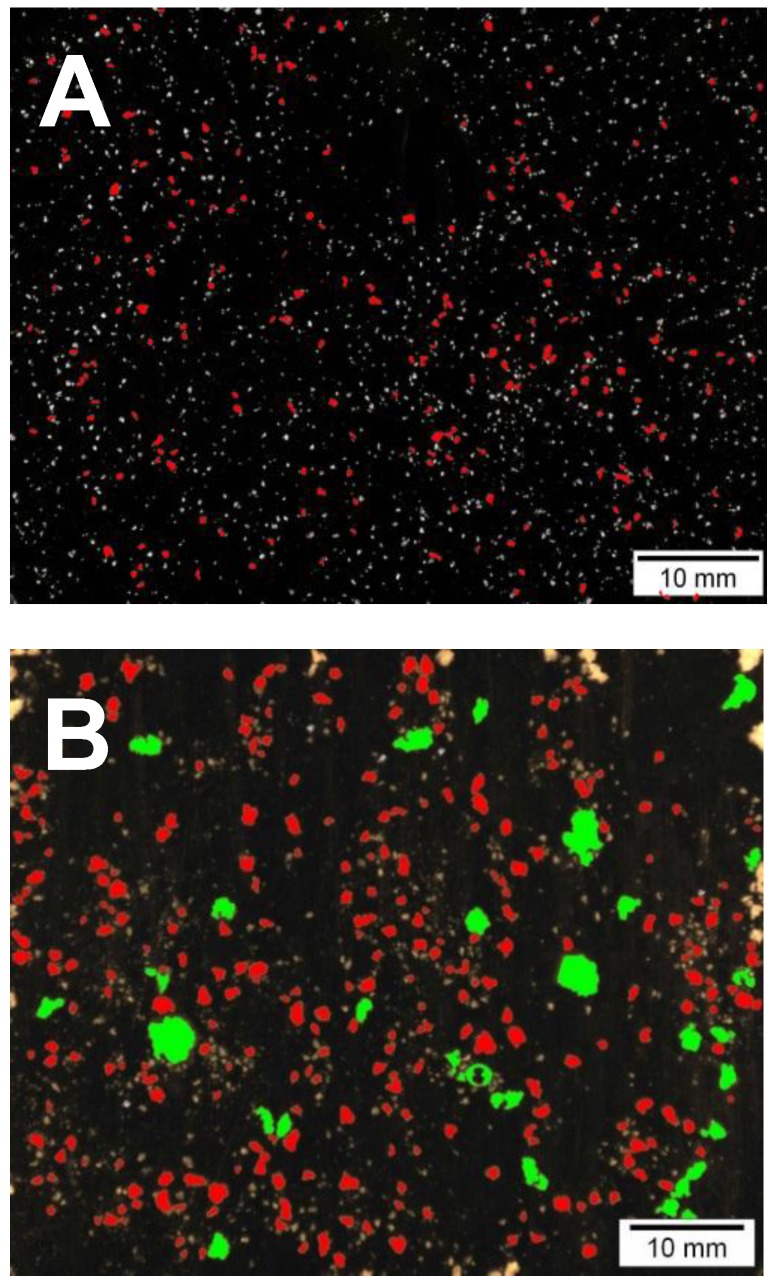
Image analysis of wheat straight dough bread (Bread 1) (**A**) and refined rye bread (Bread 6) (**B**) after chewing in mouth and *in vitro* incubation with pepsin: diameter of particles ≥ 2 mm shown green, diameter of particles < 2 mm shown red, diameter of particles < 1 mm shown grey.

After pepsin incubation described above the whole suspension was diluted with water to 200 mL and divided onto 20 Petri dishes. The particles were photographed by camera (Nikon Coolpix 5400, Nikon Corp., Tokyo, Japan). The maximum diameter and the number of particles were analysed with the AnalySIS 3.0 image analysis program (Soft Imaging System, Münster, Germany). To visualize the sizes of the different particles in the images, the large particles (diameter > 2 mm) are by image analysis shown green and the small ones (0.5–2.0 mm) red. Particles over 2 or 3 mm were considered as large particles (Figure 1). For microscopy, after the *in vitro* gastric treatment the liquid was decanted from the suspension and the residue was chemically fixed in 1% glutaraldehyde, and was embedded in agar gel and dehydrated and embedded in Historesin (Jung, Heidelberg, Germany) as recommended by the manufacturer. Sections 2 mm thick were cut with a microtome (Leica Jung RM2055, Nussloch, Germany). The sections were transferred onto glass slides and stained with 0.1% Light Green and Lugol iodine solution (0.33% I_2_ and 0.67% KI). In light micrographs of bread, protein appears green and starch dark. The samples were examined with a microscope (Olympus BH-2 microscope, Japan). Micrographs were obtained with a SensiCam PCO CCD camera (PCO, Kelheim, Germany).

### 2.6. Statistical Analysis

The statistical significance of the differences in glucose and insulin responses between the breads was assessed using the nonparametric Friedman’s test followed by Wilcoxon’s test for pairwise comparisons. To control the overall level of significance, the Bonferroni adjustment was used for pairwise comparisons of the test breads to the refined wheat bread. In all analyses, *p* values < 0.05 were considered to be statistically significant.

A multivariate analysis method, PCA (Principal Component Analysis), was used to describe the variation among the data of different breads by compressing it to the most dominant factors. The score plots demonstrated the location of the samples, and the loading plot location of measured values on the usually two-dimensional map. The map is used to show the similarities, differences and groupings of the samples and their properties. The means of measured values were used in PCA. Models were produced using SIMCA-P12.0.1 software (Umetrics Ltd, Malmö, Sweden).

## 3. Results

### 3.1. Chemical Composition and Texture of the Breads

Chemical composition, volume and acidity of the breads are presented in Table 1. Refined rye breads (55%) contained the highest amount of starch, whereas wholemeal rye bread with added bran (25.0%) had the lowest amount of starch. In the postprandial study, the bread portion size was designed to deliver 50 g of available carbohydrates; therefore, the amount of breads eaten varied from 91 g (refined rye, Bread 6) to 199 g of bread (wholemeal rye sourdough bread with rye bran, Bread 4). Wholemeal rye sourdough bread with added bran (Bread 4) contained the highest amount DF (14.5%), whereas refined wheat bread (Bread 1) and refined rye sourdough bread with gluten (Bread 7) had the lowest amount of DF (1.9% and 5.4%, respectively). The amount of DF varied from 1.9 g/consumed portion (refined wheat, Bread 1) to 28.9 g/consumed portion (wholemeal rye sourdough bread with rye bran, Bread 4).

Breads made with sourdough method had clearly smaller specific volume and harder texture as compared to breads made with straight dough method (Table 1). The volume of pan breads varied from 1.7 mL/g (wholemeal rye sourdough bread with added bran, Bread 4) to 4.5 mL/g (refined wheat, Bread 1) and those of flat breads between 1.9 and 2.2 mL/g (refined rye bread without and with gluten, Breads 6 and 7, respectively). The pH of the breads varied from around 6 (straight dough, Breads 1, 8 and 9) to around 4.5 (refined rye sourdough, Breads 5–7). TTA varied from 3.3 (refined wheat, Bread 1) to 14.8 (wholemeal rye sourdough bread with bran, Bread 4).

### 3.2. Microscopy of Refined Wheat and Wholemeal Rye Breads after in Vitro Digestion

**Figure 2:**
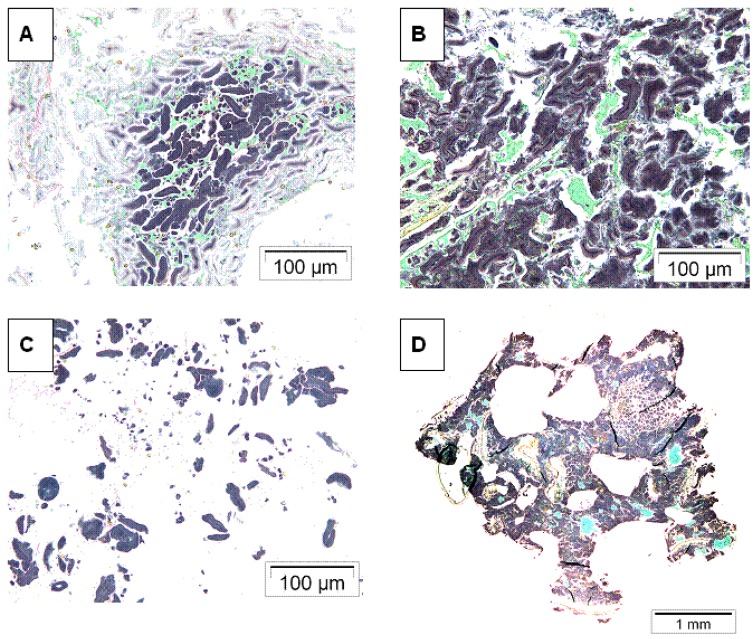
Micrographs of the residues after chewing in mouth: (**a**) wheat straight dough bread (Bread 1); and (**b**) whole meal rye sourdough bread (Bread 2). Micrographs of the residues after incubation with pepsin: (**c**) wheat straight dough bread (Bread 1); and (**d**) whole meal rye sourdough bread (Bread 2).

The micrographs of the residues after *in vitro* digestion of wheat straight dough bread (Bread 1, Figure 2A,C) and whole meal rye sourdough bread (Bread 2, Figure 2B,D) are shown in Figure 1. It was obvious that microstructure of the digesta particles of the rye breads was much less disintegrated compared to wheat breads. The difference in microstructure was seen already after chewing (Figure 2A,B) but was more pronounced after the gastric phase (Figure 2C,D). After *in vivo* chewing, the digesta particles of refined wheat bread were broken down from the edges, whereas the particles from rye sourdough bread were mainly intact. After the *in vitro* gastric step, the protein network of the wheat bread residue was extensively hydrolyzed and starch granules were freely distributed in the sample. Then again, the rye bread digesta particles resembled the original rye bread, in which aggregated starch granules form a continuous network and protein acts as filler.

### 3.3. In Vitro Particle Size Distribution of the Test Breads

The particle size distribution of the breads after *in vitro* digestion was analyzed by image analysis (Table 2). To visualize the differences in particle size in the digested material, small particles (Ø < 2 mm) were labeled red in image analysis and large particles (Ø > 2 mm) were labeled green. Image analysis in Figure 1 shows wheat straight dough bread (Bread 1, Figure 1A) and refined rye bread (Bread 6, Figure 1B) after chewing in mouth and *in vitro* incubation with pepsin. The percentage of particles over 3 mm (Ø) varied from 0.1% (refined wheat bread, Bread 1) to 7.9% (flat refined rye sourdough bread, Bread 6). The relative portion of particles over 2 mm (Ø) varied from 0.2% (refined wheat bread, Bread 1) to 16.5% (flat refined rye sourdough bread, Bread 6). The refined wheat and mixed wheat breads made with straight dough method (Breads 1, 8, 9, and 10) had the highest relative portion of particles between 0.2–2 mm (Ø), and also the average diameter of the large particles of the digested wheat breads was low. Commercial wholemeal rye bread (Bread 2,) as well as experimental refined rye breads (Breads 5–7) had higher percentage of particles over 2 or 3 mm and larger average size of particles after *in vitro* digestion than the wheat breads. Both the proportion of large particles and average particle diameter were larger in flat bread (Bread 6) in comparison to pan bread (Bread 5), and addition of gluten to refined rye sourdough bread (Bread 7) reduced the proportion of large particles.

### 3.4. Postprandial Glucose and Insulin Responses

No differences were found in glucose responses (AUC values) of the breads, but insulin responses (AUC values) of most 100% rye sourdough breads (Breads 2, 3, 5, and 6) were significantly lower than those of refined wheat bread controls (Table 3). The insulin responses of wholemeal rye sourdough bread with added bran (Bread 4) and the refined rye sourdough bread with added gluten (Bread 7) did not differ from refined wheat bread. The bread containing 60% wholemeal and 40% refined wheat flour made with straight dough method (Bread 9) had significantly higher insulin response than the refined wheat bread.

### 3.5. Correlation between Bread Properties, Particle Size, and Glucose and Insulin Response with PCA

**Figure 3:**
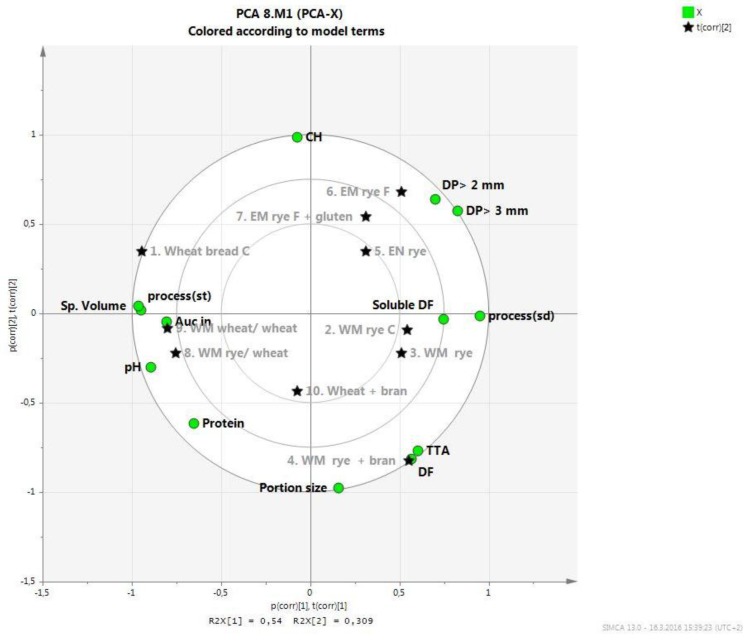
Results of PCA (Principal Component Analysis) analysis presented as Biplot picture of relation on properties of bread, particle size after *in vitro* digestion and *in vivo* response (AUC (area under the curve) values for insulin) (shown as green circles). Refined rye, whole meal rye and wheat breads are illustrated with stars, squares and hexagons, respectively. WM = wholemeal, EN = refined, C = commercial bread, F = flat bread, CH = carbohydrates, Auc in = AUC values for insulin. Number coding for breads is according to Table 1.

PCA was applied to study the relationships between chemical composition, structural features, *in vitro* disintegration and *in vivo* response of breads. Due to the non-significant statistical differences, the AUC values for glucose were not included in the PCA test. Figure 3 shows a Biplot with PC 1 plotted against PC2. The first factor explained 54% of the variation in measured properties and the second factor 31% of the variation. Wheat breads were plotted opposite to rye breads, which further were grouped to wholemeal and refined rye breads. Insulin response (AUC values) was grouped with specific volume, pH and straight dough process, indicating that samples having high pH, low density and made with straight dough process had high insulin response. The AUC values for insulin a had strong negative correlation with the number of larger particles after *in vitro* digestion and amount of soluble fiber as well as sourdough process, indicating that samples prepared with sourdough process had a high number of larger particles and lower insulin response. Total dietary fiber content, portion size or amount of carbohydrates or proteins was not correlated with the insulin response.

## 4. Discussion

The results of the present study show that bread structure and acidity influence *in vitro* digestibility and insulin responses. The glucose responses (glucose AUC values) did not show significant differences among the different breads, and thus multivariate analysis of glucose response and bread characteristics was not performed. Of the bread characteristics, low specific volume and high acidity, but, for example, not total DF content correlated with slower disintegration *in vitro* and lower insulin response *in vivo*. The lowering of insulin response as compared to refined wheat bread was linked to higher proportion of large particles of the bread after digestion *in vitro*. The result suggests that gastric particle disintegration *in vitro* could provide a tool for prediction of insulin response *in vivo*, like the glycemic index method is used to predict glycemic response. As shown by Eerdelink *et al.* [27], slower intestinal uptake of glucose from a starchy food product can result in lower postprandial insulinemia even in the absence of lower glycemic response, and provide longer-term health effects. The macro- and microstructural characteristics of breads resulting in large particles in simulated gastrointestinal digestion and consequently lower insulin index are discussed more in detail below.

### 4.1. Macrostructural Aspects

The macrostructure and texture of cereal products are known to affect their digestibility *in vivo*. Disintegration and particle size of food products during digestion is dependent on food texture and is known to influence the rate of gastric emptying and thus the glycemic responses [19]. Porous food structures, such as puffed rice and wheat, corn flakes and corn chips are known to produce high glucose and insulin responses [6,28]. In the present work, the refined wheat bread and wheat bread with 60% wholemeal flour, made with straight dough method, were easily disintegrated during *in vivo* chewing and subsequent *in vitro* pepsin incubation, as shown by the lowest number of large particles. The size of wheat bread digesta particles was typically less than 50 μm. On the other hand, the wholemeal and refined rye breads prepared by sourdough method were disintegrated *in vitro* to a much lower extent than the wheat breads, and the bread digestas contained several particles with size over 2 or 3 mm. Furthermore, the number of particles with the average diameter of particles over 2 or 3 mm correlated with AUC for insulin in PCA. Porosity provides one probable explanation for the observed differences between the wheat and rye breads. Standard wheat bread has a very porous structure, which enables salivary α-amylase to penetrate easily through the pores and disintegrate the bread matrix, whereas more dense rye sourdough breads were less susceptible for disintegration. Similarly in wheat bread increasing density has been linked to lowered digestibility [6,29]. Form of rye sourdough bread was also found to affect the *in vitro* disintegration of bread (Breads 5 and 6). The number of particles in the *in vitro* digesta was higher and average diameter higher in the flat refined rye bread in comparison to corresponding pan bread, most likely due to the larger amount of crust and the denser structure of the flat bread. Addition of gluten in rye sourdough bread (Bread 7) decreased the number of large particles *in vitro* and increased insulin response to similar value as with refined wheat bread. As in wheat bread, gluten increases porosity and reduces firmness of the rye bread crumb structure, which most probably explains the increased disintegration and insulin response of the rye bread with added gluten. Breadmaking method, straight dough or sourdough, presumably influenced bread digestibility as well. Wholemeal mixed wheat breads (60% wholemeal and 40% of refined wheat, Breads 8–10) made with straight dough method had bigger volume and softer texture, showed less large digesta particles than the 100% rye sourdough breads, and comparable or higher insulin response than refined wheat bread. However, the breads made by straight dough process contained both refined and wholegrain flour, so the results are not directly comparable to 100% sourdough wholegrain rye bread.

### 4.2. Microstructural Aspects

Microstructure of food is known to influence substrate-enzyme interactions and thus susceptibility of nutrients to gastrointestinal enzymes. Generally in wheat bread making, gluten forms the continuous phase of the bread matrix, whereas starch granules form the continuous phase of rye breads [11,30]. In addition, in the tested rye sourdough breads the continuous bread matrix was composed of swollen starch granules, leached amylose and swollen cell walls, thus making proteins probably less susceptible for hydrolysis under conditions mimicking gastric environment. The sourdough process also changes the microstructure of rye bread by solubilization of proteins and DF, mainly arabinoxylan that is the main water binding substance in rye sourdough [30]. These changes by sourdough are partly acidity-induced, either directly by acid or by induction of endogenous xylanases and proteases by acidic environment. Acid and its effect on proteins and DF has been proposed to affect the glycemic responses to breads [15], and besides microstructural changes, it has been suggested that one reason for the acid-induced reduced postprandial responses could also be delayed gastric emptying rate due to organic acids produced by sourdough microflora [31,32].

In the case of wheat bread microstructure, pieces of bread after mastication were large, and gluten network was dominating and binding the dispersed individual starch granules. However, under conditions mimicking gastric digestion, the pieces of standard wheat bread were easily broken down since pepsin has access to protein phase surrounding the starch granules. This was also supported by the microscopy analysis of wholemeal rye sourdough bread were the continuous phase of swollen starch granule aggregates protected starch and protein against enzymatic hydrolysis. This can be one reason why disintegration of rye sourdough bread is reduced as compared to wheat bread. Adding gluten to rye sourdough bread (Bread 7) might also have changed the microstructure and continuous phase of rye bread with subsequent increase in disintegration by pepsin during *in vitro* digestion. Interestingly, the rye sourdough bread with added bran (Bread 4) also showed increased insulin response that did not differ from refined wheat bread. The mechanism behind the result remains to be studied in more detail, but possibly the bran addition physically disrupted the dense structure of rye sourdough bread. Bran has typically high degree of insoluble components, especially insoluble dietary fiber that might have disrupted the rye crumb structure consisting of viscous network made of soluble arabinoxylan and starch and thus making bread components more susceptible for digestive enzymes. Generally, in a view of these results and hypothesis, it was rather unexpected that only the insulin responses but not those of glucose were lower to rye sourdough breads than to wheat breads. However, also previous studies have demonstrated that sourdough rye breads or other foods rich in starch products display low insulinemic response, regardless of their glucose response [27,33].

### 4.3. Limitations of the Study

Combining the data of several *in vivo* experiments carried over several years brings an element of variability to the results. Besides natural variation between the studies performed at different times, the subjects for testing the commercial breads (Breads 1 and 2) were young men and women and in the other experiments older women. However, all subjects were healthy and the age and gender differences in the subject groups are not expected to cause major impact on the results. There were also some weaknesses in the dataset for the comparison of the bread characteristics. For example, bread of 100% rye flour with straight dough process was not available to be compared to the corresponding sourdough rye bread. Nevertheless, the breads studied provided enough variance with respect to structural parameters to suggest that disintegration of structure during early phases of gastrointestinal digestion might be one factor contributing to postprandial control of glucose metabolism.

## 5. Conclusions

Relationships were shown between chemical and structural properties of rye and wheat breads, their *in vitro* gastric disintegration and *in vivo* insulin, but not glucose responses. High relative proportion of large sized particles after chewing and *in vitro* gastric digestion was associated with low postprandial insulin responses, suggesting that analysis of structural disintegration is a complementary tool in predicting postprandial physiology.

## Figures and Tables

**Table 1:** Characteristics of rye and wheat breads (chemical composition expressed g/100 g fresh weight). Standard deviation in chemical composition data was less than 5%.

	Bread type	Baking Process	Portion Size (g)	Sugars	Available Carbohydrate	Dietary Fiber	Soluble Dietary Fiber	Protein	Fat	Ash	Moisture	pH	TTA	Bread Volume, mL/g
1	Refined wheat	Straight dough	99.2	2.0	50.4	1.9	0.5	8.6	4.9	1.4	33.7	6.0	3.3	4.5 ± 0.1
2	Wholemeal rye (Commercial)	Sourdough	134.8	5.0	37.2	9.1	1.6	6.1	1.4	nd ^a^	41.1	4.3	10.9	0.9 ± 0.04
3	Wholemeal rye	Sourdough	142.9	0.3	35.0	10.7	3.0	7.8	5.5	1.6	40.8	4.9	10.2	1.8 ± 0.20
4	Wholemeal rye + bran	Sourdough	199.2	0.2	25.1	14.5	2.4	8.4	5.1	2.4	45.2	5.1	14.8	1.7 ± 0.20
5	Refined rye	Sourdough	112.1	0.4	44.6	5.7	2.1	4.1	0.7	1.0	43.1	4.4	5.1	2.2 ± 0.20
6	Refined rye (flat)	Sourdough	90.6	1.1	55.2	6.4	2.4	3.7	0.4	1.2	31.5	4.7	5.5	1.9 ± 0.10
7	Refined rye + gluten (flat)	Sourdough	100.6	1.0	49.7	5.4	2.2	4.4	0.4	1.1	37.2	4.5	4.9	2.2 ± 0.10
8	Wholemeal rye/ wheat	Straight dough	137.7	1.5	36.3	7.1	2.0	12.9	2.6	1.8	39.4	6.1	4.3	3.8 ± 0.03
9	Wholemeal wheat/ wheat	Straight dough	135.9	1.8	36.8	6.0	0.8	8.6	13.7	nd	38.3	5.9	5.3	4.2 ± 0.05
10	Refined Wheat + fermented bran	Straight dough	162.3	2.2	30.8	10.6	1.1	8.0	4.4	2.0	46.9	5.5	10.3	3.3 ± 0.03

^a^ not determined.

**Table 2:** Particle size distribution (%) of the test breads after *in vitro* digestion of bread pieces (bread amount dosed based on 1 g potentially available carbohydrates).

Bread	Distribution of Digesta Particles (%)			Average Diameter of Digesta Particles (DP ^a^)	
	0.2–2 mm	>2 mm	>3 mm	>2 mm	>3 mm
1. Refined wheat (commercial) (st) ^b^	99.8	0.2	0.1	2.6 (±0.2)	3.5 (±0.3)
2. Wholemeal rye (commercial) (sd) ^c^	94.4	5.6	2.5	3.6 (±0.2)	5.1 (±0.2)
3. Wholemeal rye (sd)	nd ^d^	nd	nd	nd	nd
4. Wholemeal rye+bran (sd)	nd	nd	nd	nd	nd
5. Refined rye (sd)	93.1	6.9	2.2	2.9 (±0.1)	4.0 (±0.2)
6. Refined rye, flat (sd)	83.5	16.5	7.9	3.4 (±0.0)	4.4 (±0.2)
7. Refined rye+gluten, flat (sd)	86.6	13.4	4.8	3.3 (±0.1)	5.0 (±0.3)
8. Wholemeal rye/wheat (st)	97.4	2.6	0.5	2.7 (±0.2)	3.8 (±0.2)
9. Wholemeal wheat/wheat (st)	97.6	2.4	0.2	2.4 (±0.01)	3.4 (±0.3)
10. Refined Wheat + fermented bran (st)	94.9	5.1	0.8	2.5 (±0.1)	3.5 (±0.2)

^a^ DP = Average diameter of over 2 mm or over 3 mm particles; ^b^ st = straight dough process; ^c^ sd = sourdough process, ^d^ nd = not determined.

**Table 3:** The postprandial incremental blood glucose and insulin responses of the breads determined as areas under the curve (AUC) over 180 min. The values are shown as means with standard deviation. Statistical significance of differences within each series was assessed using the nonparametric Friedman´s test. In case a significant difference was observed, Wilcoxon´s test for pairwise comparisons was made to compare the other samples to refined wheat bread. In bread coding “sd” stands for sourdough and “st” for straight dough.

	Bread	Glucose Area	Statistics ^1,2^	Insulin Area	Statistical Significance ^1,2^
Series 1 (*n* = 17)					
	1. Refined wheat (commercial)	98 ± 91		3342 ± 1600	
	2. Wholemeal rye (commercial) (sd)	79 ± 63	*P* = 0.709 ^2^	2086 ± 889	*P* = 0.001 ^2^
Series 2 (*n* = 12)					
	Refined wheat	90 ± 49	*P* = 0.940 ^1^	3289 ± 1105	*P* = 0.001 ^1^
	3. Wholemeal rye (sd)	71 ± 42		2469 ± 954	*P* = 0.031 ^2^
	4. Wholemeal rye+bran (sd)	103 ± 120		2758 ± 1013	*P* = 0.166 ^2^
	5. Refined rye (sd)	95 ± 84		2326 ± 737	*P* = 0.006 ^2^
Series 3 (*n* = 12)					
	Refined wheat	143 ± 88	*P* = 0.717 ^1^	3661 ± 1476	*P* = 0.013 ^1^
	6. Refined rye, flat (sd)	190 ± 95		2811 ± 844	*P* = 0.020 ^2^
	7. Refined rye+ gluten, flat (sd)	191 ± 97		3212 ± 1368	*P* = 0.628 ^2^
Series 4 (*n* = 12)		121 ± 90	*P* = 0.127 ^1^	3658 ± 1656	*P* = 0.627 ^1^
	8. Wholemeal rye/wheat (st)	109 ± 94		3600 ± 1365	
	10. Refined Wheat + fermented bran (st)	74 ± 41		3758 ± 1512	
Series 5 (*n* = 12)					
	Refined wheat	100 ± 58		3435 ± 1535	
	9. Wholemeal wheat/wheat (st)	98 ± 56	*P* = 0.936 ^2^	4173 ± 1722	*P* = 0.018 ^2^

^1^ Friedman’s test and ^2^ Wilcoxon’s test with Bonferroni corrections in series 2 and 3.

## Supplementary Materials

Supplementary materials are available online at www.mdpi.com/link.
