# Supplementary material for: Distinct Characteristics of Rye and Wheat Breads Impact on Their in Vitro Gastric Disintegration and in Vivo Glucose and Insulin Responses
Source: Foods. 2016 Mar 25;5(2):24. doi: 10.3390/foods5020024 (PMC5302334; doi:10.3390/foods5020024)
Supplement: Supplementary file 1 [file foods-05-00024-s001.pdf]

Supplementary Materials: Distinct Characteristics of Rye and Wheat Breads Impact on Their *in Vitro* Gastric Disintegration and *in Vivo* Glucose and Insulin Responses

Nordlund Emilia *, Katina Kati, Mykkänen Hannu and Poutanen Kaisa

Table S1. Bread recipes for experimental breads (% of dough weight). Breads: 1 Refined wheat (st) 2, Wholemeal rye (Commercial) (sd), 3 Wholemeal rye (sd), 4 Wholemeal rye + bran (sd), 5 Refined rye (sd), 6 Refined rye (flat) (sd), 7 Refined rye + gluten (flat) (sd), 8 Wholemeal rye/ wheat (st), 9 Wholemeal wheat/ wheat (st), 10 Wheat + fermented bran (st) (sd = sourdough; st = straight dough).

Raw Materials	Bread No.							
	3	4	5	6	7	8	9	10
Wholgrain rye flour	43	24				28		
Wholegrain wheat flour							31	
Refined rye flour			40	40	39			
Refined wheat flour						20	21	28
Wheat bran								7
Fresh yeast	0.8	0.9	0.9	0.9	0.9	1.56	1.7	
Dried yeast								0.53
Rye bran		17						
Water	21	26	22	22	21	42	38	14
Salt	0.6	0.6	0.6	0.6	0.6	1.1	0.9	0.53
Gluten					1.2	6	6.6	
Sourdough of wholgrain rye flour (A) [a]	35	32						
Sourdough of refined rye flour (B) [b]			37	37	37			
Fermented wheat bran (C) [c]								32
Sugar								
Fat						0.71	0.7	2.1
Enzymes [d]								0.025

[a] Wholemeal rye sourdough: wholegrain rye flour 37% (of dough weight), salt 0.4%, Lb. brevis 0.37%, Lb. plantarum 0.37%, water 62%, fermentation time 22 h at 30 °C; [b] Refined rye sourdough: refined rye flour 37% (of dough weight), salt 0.4%, Lb. brevis 0.036%, Lb. plantarum 0.036%, water 62%, fermentation time 23 h at 30 °C; [c] Bran fermentation: wheat bran 22% (of dough weight), dried yeast 2.8%, water 78%, fermentation time 16 h at 25 °C; [d] Grindamyl max life (Danisco, Denmark) 0.01% flour weight, + Lipopan 50 BG 0.004% (Novo Nordisk, Denmark), + Pentopan mono BG 0.01% (Novo Nordisk, Denmark).

Table S2. Breadmaking process for the experimental breads. Breads: 1 Refined wheat (st) 2, Wholemeal rye (Commercial) (sd), 3 Wholemeal rye (sd), 4 Wholemeal rye + bran (sd), 5 Refined rye (sd), 6 Refined rye (flat) (sd), 7 Refined rye + gluten (flat) (sd), 8 Wholemeal rye/ wheat (st), 9 Wholemeal wheat/ wheat (st), 10 Wheat + fermented bran (st) (sd = sourdough; st = straight dough).

Bread No.	3	4	5	6	7	8	9	10
Bread Type	pan	pan	pan	flat	flat	pan	pan	pan
Mixing time/min	2 + 2	2 + 2	2 + 2	2 + 2	2 + 2	3 + 5	3 + 5	3 + 6
Resting time/min	45	45	45	2	2	30	30	20
Resting temperature/ ℃ and humidity%	28/70	28/70	28/70	20	20	28/70	28/75	29/76
Proofing time/min	45	70	50	45	45	55	60	55
Proofing temperature/ ℃ and humidity%	35/70	35/70	35/70	35/50	35/50	37/80	37/75	37/80
Loaf size/g	600	600	400	120	120	500	400	400
Baking time/min	50	50	40	20	20	35	20	20
Baking temperature/ ℃	240–220	240–220	240–220	250	250	240–220	230	200
